# Analysis of current status and influencing factors of participation in medication safety behaviors among older adult patients with chronic co-morbidities: based on the COM-B model

**DOI:** 10.3389/fpubh.2025.1695668

**Published:** 2025-10-15

**Authors:** Yuxin Li, Lin He, Tianxia Zhao, Ping Dai, Yanhong Wen, Yuting Fan, Qin Lin, Jijun Wu

**Affiliations:** ^1^Department of Nursing, Deyang People’s Hospital, Deyang, Sichuan, China; ^2^Department of Respiratory and Critical Care, Deyang People’s Hospital, Deyang, Sichuan, China; ^3^Department of Cardiology, Deyang People’s Hospital, Deyang, Sichuan, China; ^4^Shulan International Medical College, Zhejiang Shuren University, Hangzhou, Zhejiang, China

**Keywords:** chronic disease comorbidity, older adults, participation in medication safety behaviors, nursing, COM-B model

## Abstract

**Background:**

With the acceleration of global aging, the prevention and control of chronic disease comorbidity have become increasingly challenging, emerging as a significant global public health issue. Patients with multiple coexisting conditions often face complex treatment regimens and multiple medications, posing significant challenges to their participation in medication safety behaviors. Individual health behaviors are influenced by knowledge, beliefs, and social environment, among other factors. Therefore, this study employs the COM-B model to analyse the factors influencing participation in medication safety behaviors among older adult patients with chronic coexisting conditions, aiming to provide insight into participation in medication safety behaviors.

**Methods:**

This cross-sectional study employed convenience sampling to survey 335 older adult patients with chronic disease comorbidity at a Grade A tertiary hospital in Sichuan Province, China, from July to December 2024. The survey employed a general information questionnaire, the participation in medication safety behaviors scale, the health literacy scale, the medication belief scale, the family APGAR questionnaire, and the social network scale. Descriptive analysis, univariate analysis, correlation analysis, and multiple linear regression analysis were conducted using SPSS 26.0 software.

**Results:**

The mean participation in medication safety behaviors score among 335 older adult patients with chronic comorbidities was 101.36 ± 16.68. Correlation analysis revealed that the total score and individual dimension scores of participation in medication safety behaviors among older adult patients with chronic comorbidities were positively correlated with the total scores of health literacy, medication belief, family function, and social network (*r* = 0.347–0.703, *p* < 0.01). Multivariate linear regression analysis revealed that education level, drug concerns, disease knowledge level, health literacy, medication belief, family function, and social network were significant predictors in the regression equation (*p* < 0.05), accounting for 75.5% of the total variance.

**Conclusion:**

Chinese older adult patients with chronic disease comorbidity demonstrate moderate participation in medication safety behaviors. Education level, drug concerns, disease knowledge level, health literacy, medication belief, family function, and social network are key determinants influencing medication safety behaviors among this population. The COM-B model provides a framework for explaining low participation in medication safety behaviors among older adult patients with chronic disease comorbidities and guides the development of targeted health intervention strategies.

## 1 Introduction

Chronic disease comorbidity refers to an individual suffering from two or more chronic noncommunicable diseases simultaneously (1). As the global population continues to age, this phenomenon is becoming increasingly prevalent among the older adults. Statistics indicate that approximately 40 to 56% of individuals aged 65 and older worldwide experience chronic disease comorbidity (2). As the world’s most populous developing nation, China has entered a stage of deep aging, where chronic disease comorbidity is particularly pronounced. The prevalence of comorbidity among the older adult population reaches approximately 60% (3). Chronic disease treatment primarily relies on medication. Compared to individuals with a single chronic condition, those with comorbidity often require multiple concurrent medications (4). With age-related physiological decline, medication-related risks significantly increase, making medication safety an increasingly prominent concern (5). A meta-analysis revealed that the rate of polypharmacy among adults worldwide is 37%, while the rate among the older adult population reaches as high as 45% (6). The direct consequence of polypharmacy is an elevated incidence of medication-related adverse events (7). Previous studies indicate that medication errors in polypharmacy settings can reach up to 75%, with adverse drug reactions occurring 76% more frequently than in monotherapy (8). Consequently, medication safety has emerged as a significant global public health concern.

Medication safety refers to the process of protecting patients from harm while maximizing therapeutic benefits by preventing medication errors (9). Participation in medication safety behaviors refers to the process by which patients proactively adopt a series of cognitive, decision-making, and operational measures to reduce medication-related risks and ensure the safety and efficacy of drug therapy. Its core objective is to prevent medication errors and promote rational drug use (10). Previous studies indicate that older adult patients with chronic comorbidities exhibit poor engagement in medication safety behaviors, influenced by multiple factors such as gender, age, educational attainment, number of medications, and dosing frequency (5, 11). Unsafe medication practices not only directly lead to serious consequences like adverse drug events and treatment failure, severely compromising physical and mental health and quality of life, but also result in wasted healthcare resources and a sharp increase in family financial burdens (12, 13). Therefore, thoroughly examining the factors influencing medication safety behaviors among older adult patients with chronic comorbidities is crucial for preventing medication errors, improving patient outcomes, and developing scientifically sound intervention strategies to enhance medication safety and promote rational drug use.

**Figure 1:**
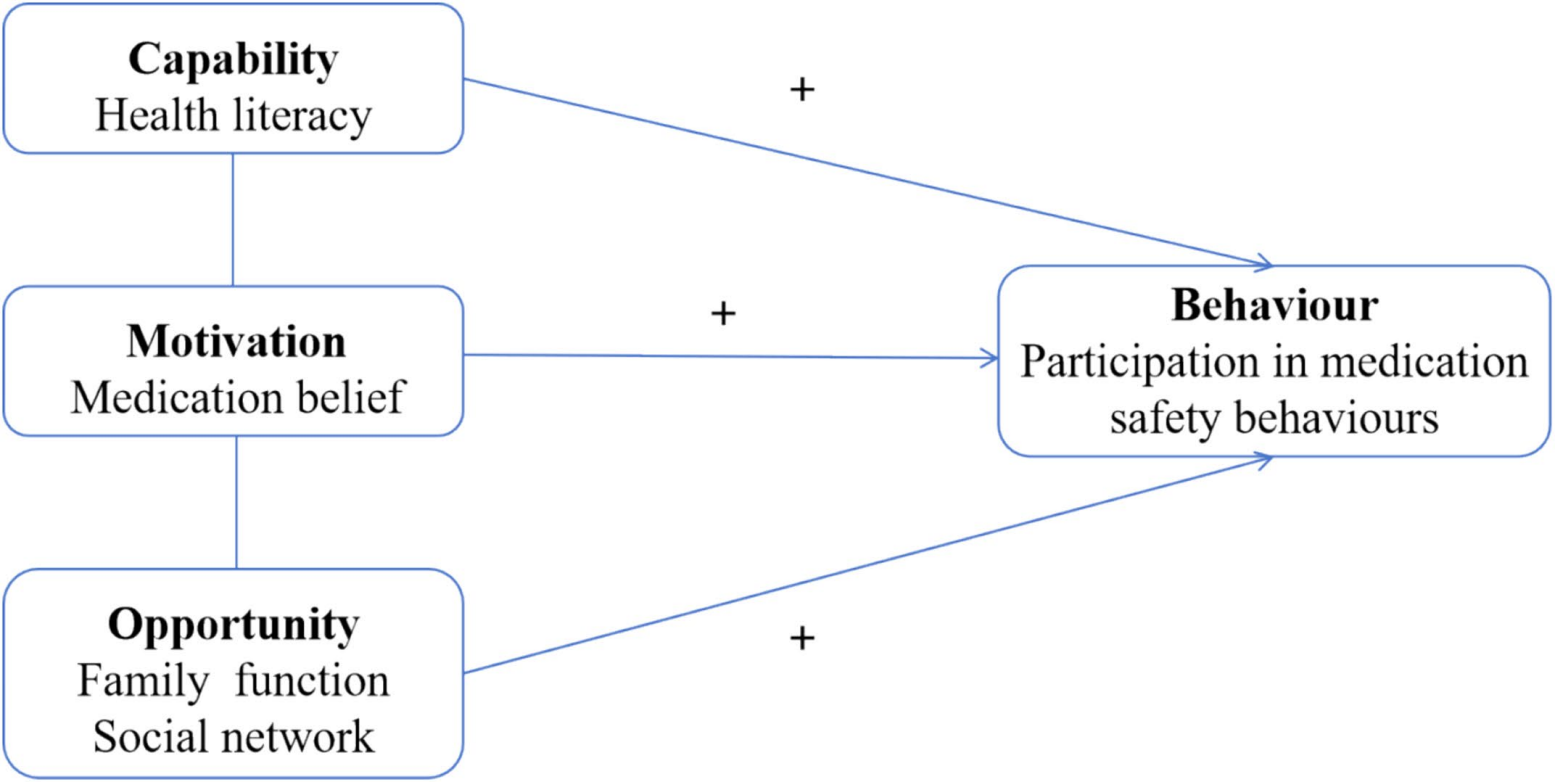
Theoretical framework of this study.

Previous studies on participation in medication safety behaviors among patients with chronic diseases have primarily relied on single theoretical models, failing to comprehensively consider the combined influence of multidimensional factors, such as individual cognition, social environment, health knowledge, and beliefs. The Capability-Opportunity-Motivation-Behavior (COM-B) model, as a key theoretical framework for studying health-related behaviors, systematically analyses behavioral determinants across three dimensions: capability, opportunity, and motivation. It is well-suited for identifying critical factors that influence complex health behaviors (14). Within this model, “capability” refers to the psychological and physical abilities required for an individual to perform a specific behavior. Health literacy reflects a patient’s fundamental ability to access, understand, and apply medication information, serving as a core element within the “Capacity” dimension (15). “Motivation” encompasses all conscious or unconscious internal drivers that initiate, guide, and sustain behavior. Medication beliefs encompass an individual’s cognitive perceptions and attitudes regarding the necessity, efficacy, and concerns associated with drug therapy (16), constituting a vital component of the “Motivation” dimension. “Opportunity” refers to all environmental factors external to the individual that enable or facilitate behavior. Family function and social networks emphasize the role of tangible resources and environmental conditions, including family support and social relationships, in promoting medication adherence (17, 18). They can be incorporated into the “opportunity” dimension. The COM-B model has been widely applied in various fields, including chronic disease self-management and health promotion, with its validity and reliability extensively validated (19, 20). Therefore, this study employs the COM-B model to conduct an in-depth analysis of factors influencing participation in medication safety behaviors among older adult patients with chronic comorbidities across three dimensions: capacity (health literacy), opportunity (family function and social network), and motivation (medication belief) (see Figure 1). This provides a theoretical foundation for developing comprehensive, scientifically grounded, targeted interventions to enhance participation in medication safety behaviors among this population.

## 2 Methods

### 2.1 Study design

This study is a cross-sectional study.

### 2.2 Participants

**Figure 2:**
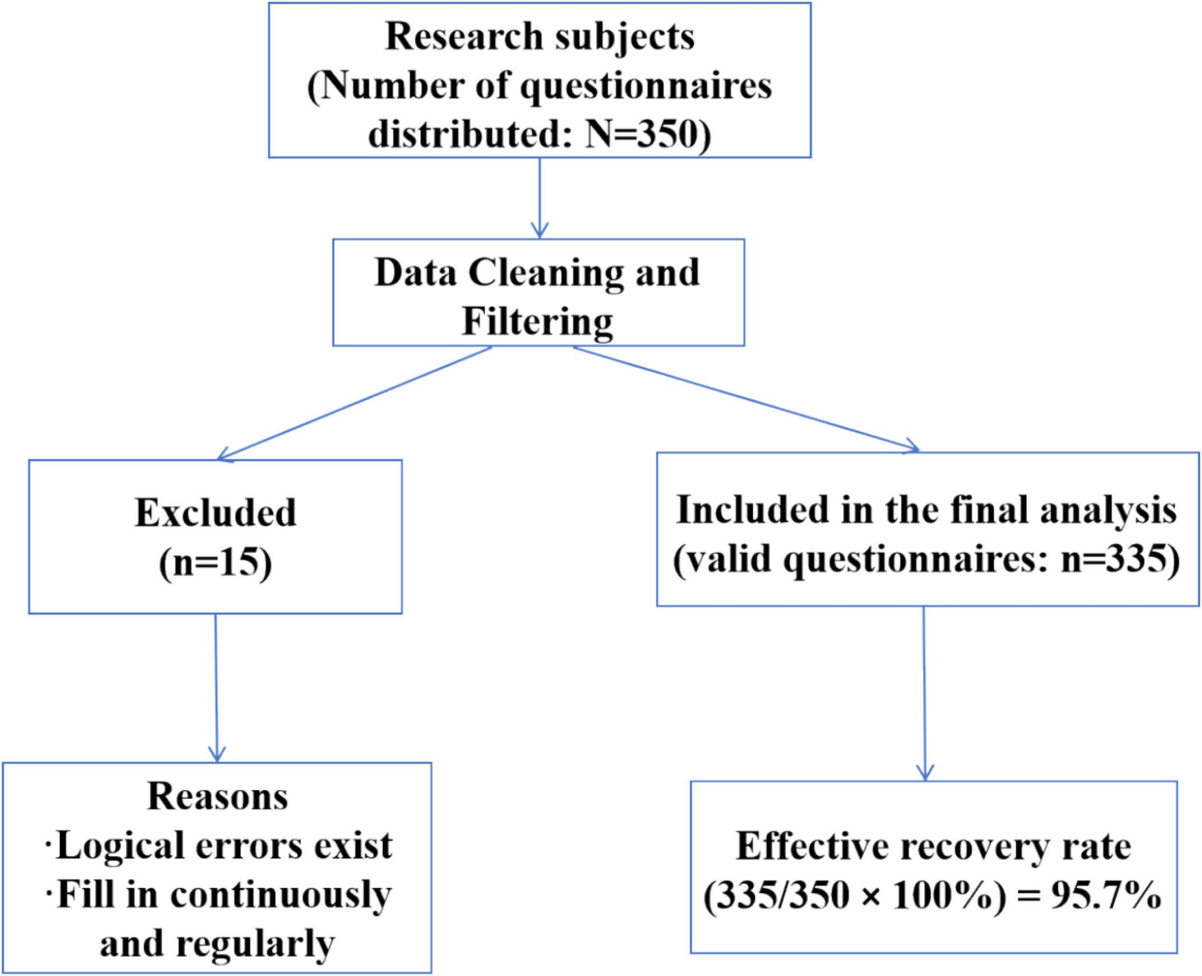
Participant flowchart.

Using convenience sampling, 335 older adult patients with chronic disease comorbidity were selected from a Grade A tertiary hospital in Sichuan Province, China, between July and December 2024. Inclusion criteria: (1) Diagnosed with at least two chronic diseases (including hypertension, diabetes, coronary heart disease, stroke, chronic obstructive pulmonary disease, asthma, hyperlipidemia, chronic kidney disease, chronic hepatitis B, chronic gastritis, etc.) at a secondary-level or higher hospital; (2) Age ≥65 years; (3) Informed consent and voluntary participation in this study. Exclusion criteria: (1) Impaired consciousness or significant cognitive impairment preventing cooperation with the survey; (2) Hearing or speech impairment preventing everyday communication. Based on the sample size formula *n = (u_1 -α/2_ σ/δ)*^2^ (21), with a significance level α = 0.05, *u_1 -α/2_* = 1.96, where σ is the standard deviation and *δ* is the permissible error. Based on preliminary survey results, the standard deviation σ for medication safety behaviors among older adult patients with chronic comorbidities was determined to be 0.63. With a tolerance error δ set at 0.1, the sample size n was calculated as (1.96 × 0.63/0.1)^2^ = 153. Accounting for a 20% non-response rate, the study’s planned sample size was set at 192 cases. Given sample accessibility, the number of eligible patients exceeded projections during the recruitment period. Consequently, 335 subjects were ultimately enrolled, meeting the sample size requirement. The subject flow diagram is presented in Figure 2.

### 2.3 Measures

#### 2.3.1 General information questionnaire

A self-designed general information questionnaire was used, covering gender, age, marital status, number of children, education level, place of residence, medical payment methods, average monthly family income, living situation, daily sleep duration, family members supervising medication intake, drug concerns, frequency of receiving medication safety education, number of chronic diseases, disease duration, disease knowledge level, years of medication, number of medications, frequency of medication, has a drug-related adverse event occurred.

#### 2.3.2 Participation in medication safety behaviors scale

The participation in medication safety behaviors scale was independently developed by Chinese researchers Feng et al. (22) based on the cultural and social context of the Chinese population. Its development process followed standard procedures for constructing psychological measurement tools. It is primarily used to assess the behaviors and capabilities of patients with chronic diseases in adhering to their medication regimens. The scale comprises four dimensions—medication knowledge, pharmaceutical belief, participation in medication decisions, and medication self-management—consisting of a total of 33 items. It employs a 5-point Likert scale, with responses ranging from “completely unaware” to “very knowledgeable,” scored from 1 to 5 points, respectively. The total score ranges from 33 to 165 points, with higher scores indicating stronger capabilities in medication safety participation. The scale demonstrates good reliability and validity. In this study, the Cronbach’s *α* coefficient for the scale was 0.925.

#### 2.3.3 Health literacy scale

The health literacy scale was developed by Chung et al. (23) and adapted into Chinese by Li et al. (24). The adaptation process followed Brislin’s translation-back-translation standard procedure, primarily used to assess health literacy levels among older adults. This unidimensional scale comprises 10 items. It employs a 5-point Likert scale, scoring from 1 (strongly disagree) to 5 (strongly agree), yielding a total score ranging from 10 to 50. Higher scores indicate greater health literacy. The scale demonstrates good reliability and validity. Confirmatory factor analysis indicates a well-fitted unidimensional structure, with a TLI of 0.973, CFI of 0.982, and Cronbach’s *α* coefficient of 0.945. In this study, the Cronbach’s α coefficient for the scale was 0.842.

#### 2.3.4 Medication belief scale

The medication belief scale was developed by Horne et al. (25) and adapted into Chinese by Kang et al. (26). The adaptation process followed Brislin’s translation-back-translation standard procedure. This scale is primarily used to assess patients’ levels of medication belief. It is unidimensional and consists of 10 items. It employs a 5-point Likert scale, with responses ranging from “strongly disagree” (1 point) to “strongly agree” (5 points), yielding a total score between 10 and 50. Higher scores indicate stronger medication belief. The scale demonstrates good reliability and validity, with a Cronbach’s *α* coefficient of 0.923. In this study, the Cronbach’s α coefficient for the scale was 0.813.

#### 2.3.5 Family APGAR questionnaire

The family APGAR questionnaire was developed by Smilkstein (17) and adapted into Chinese by Lü et al. (27). The adaptation process followed Brislin’s translation-back-translation standard procedure. It is primarily used to assess an individual’s family functioning. The questionnaire consists of five items across five dimensions: adaptability, intimacy, growth, cooperation, and emotional bonding. It employs a 3-point Likert scale, scoring from 0 to 2 points for responses ranging from “rarely” to “frequently.” The total score ranges from 0 to 10 points, with higher scores indicating better family functioning. The questionnaire demonstrates good reliability and validity, and is widely used in research assessing family functioning among patients with chronic diseases. In this study, the Cronbach’s *α* coefficient for this questionnaire was 0.893.

#### 2.3.6 Social network scale

The social network scale was developed by Bae et al. (18) in 2020 and revised for Chinese adaptation by Liu et al. (28). The Chinese adaptation followed Brislin’s translation-back-translation standard procedure and is primarily used to assess the frequency and satisfaction of social participation among older adults. The scale comprises four dimensions: face-to-face interactions with family, face-to-face interactions with friends, non-face-to-face interactions with family via phone, mail, or email, and non-face-to-face interactions with friends via phone, mail, or email. It consists of a total of 8 items. Social participation satisfaction is measured using a 4-point Likert scale, with responses ranging from “very dissatisfied” (1 point) to “very satisfied” (4 points). The total score ranges from 0 to 64 points, where higher scores indicate greater satisfaction with social participation among older adults. The scale demonstrates good reliability and validity, with a Cronbach’s *α* coefficient of 0.859. In this study, the Cronbach’s α coefficient for the scale was 0.877.

### 2.4 Data collection

This study employed a questionnaire survey method. Before the formal survey, researchers provided standardized training to five survey administrators. Training covered core concepts addressed in the questionnaire, completion requirements, and uniform instructions. Following training, administrators strictly selected research subjects according to inclusion and exclusion criteria. Before the survey, standardized instructions were used to thoroughly explain the study’s objectives, significance, completion methods, and precautions to participants. After obtaining informed consent and signing the consent form, paper questionnaires were distributed. For participants unable to complete the questionnaire independently or with lower educational attainment, interviewers read each question aloud, recorded responses verbatim, and verified the information. During completion, interviewers avoided prompting or interfering with participants. Upon completion, questionnaires were collected immediately by investigators, who promptly reviewed and cross-checked responses. Any omissions were addressed on-site by requesting participants to supplement their answers. A total of 350 questionnaires were distributed. After excluding 15 with logical errors or systematically filled patterns, 335 valid responses were recovered, yielding a 95.7% valid response rate.

### 2.5 Statistical methods

Statistical analysis was performed using SPSS 26.0. Normally distributed continuous variables were described using mean ± standard deviation, while categorical variables were described using frequency and proportion. Independent t-tests or one-way ANOVA were used to compare the effects of general characteristics on participation in medication safety behaviors scores among older adult patients with comorbid chronic conditions. Pearson correlation analysis was used to explore the associations between participation in medication safety behaviors and health literacy, medication belief, family care, and social network among older adult patients with chronic disease comorbidity. Multiple linear regression analysis was employed to identify factors influencing participation in medication safety behaviors in this population. *p* < 0.05 was considered statistically significant.

### 2.6 Ethical considerations

This study adheres to the Declaration of Helsinki and was approved by the Ethics Committee of Deyang People’s Hospital (Approval No. 2022-04-010-K01). Prior to the survey, the research objectives and significance were explained to participants, along with assurances regarding the anonymity of their responses, the exclusive use of the data for this study, and complete confidentiality. Informed consent was obtained, and the principles of confidentiality, non-maleficence, and beneficence were strictly observed. All subjects voluntarily participated.

## 3 Results

### 3.1 Common method bias

This study used self-report questionnaires, which may introduce some common method bias. To enhance rigor, Harman’s single-factor analysis was applied through an unrotated exploratory factor analysis. The results showed 15 factors with eigenvalues greater than 1. The first factor explained only 23.82% of the variance, below the 40% threshold. Therefore, no significant common method bias is present in this study.

### 3.2 General characteristics of older adult patients with chronic disease comorbidities

Among the 335 older adult patients with chronic disease comorbidities in this study, males accounted for 49.6% and females for 50.4%. The majority were aged 65 to <75 years (62.7%), and most were married (76.1%). Additional general characteristics are presented in Table 1.

**Table 1:** Univariate analysis of participation in medication safety behaviors among older adult patients with chronic disease comorbidities.

Items	*N* (%)	Mean ± SD	*t/F*	*p*
Gender			0.409	0.683
Male	166 (49.55)	101.73 ± 17.25		
Female	169 (50.45)	100.99 ± 16.15		
Age (years)			1.943	0.145
65 ~ <75	210 (62.69)	102.72 ± 17.67		
75 ~ <85	109 (32.54)	98.89 ± 15.15		
≥85	16 (4.77)	100.25 ± 11.01		
Marital status			2.184	0.030
Married	255 (76.12)	102.47 ± 16.77		
Single/Divorced/Widowed	80 (23.88)	97.83 ± 15.99		
Number of children			3.006	0.051
1	93 (27.76)	103.51 ± 20.06		
2 ~ 3	206 (61.49)	101.41 ± 15.25		
≥4	36 (10.75)	95.53 ± 2.29		
Education level			59.192	<0.001
Elementary and below	176 (52.54)	94.35 ± 14.01		
Junior	102 (30.45)	104.45 ± 12.33		
High school and above	57 (17.01)	117.47 ± 18.33		
Place of residence			3.975	<0.001
Towns	171 (51.04)	104.83 ± 17.70		
Countryside	164 (49.96)	97.74 ± 14.76		
Medical payment methods			16.822	<0.001
Urban medical insurance	147 (43.88)	107.05 ± 17.21		
Rural medical insurance	167 (49.85)	97.13 ± 15.36		
Self-pay	21 (6.27)	95.14 ± 9.95		
Average monthly family income (yuan)			17.767	<0.001
<1,000	80 (23.88)	91.98 ± 16.03		
1,000 ~ 3,000	161 (48.06)	101.42 ± 14.68		
3,001 ~ 5,000	68 (20.30)	108.92 ± 16.37		
>5,000	26 (7.76)	109.42 ± 17.09		
Living situation			3.383	0.018
Live alone	40 (11.94)	98.78 ± 16.67		
Living with spouse only	151 (45.07)	102.63 ± 17.32		
Living with children and spouse	128 (38.21)	102.13 ± 15.72		
Other	16 (4.78)	89.24 ± 13.97		
Daily sleep duration (h)			−0.148	0.883
≤6	71 (21.19)	101.10 ± 16.81		
>6	264 (78.81)	101.43 ± 16.68		
Are family members supervising medication intake			2.214	0.111
Frequently	64 (19.10)	104.78 ± 14.90		
Occasionally	181 (54.03)	99.81 ± 15.09		
Never	90 (26.87)	102.03 ± 20.33		
Drug concerns			57.937	<0.001
Not at all concerned	57 (17.01)	85.54 ± 14.14		
Somewhat concerned	84 (25.07)	95.30 ± 13.11		
Very concerned	130 (38.81)	105.28 ± 12.68		
Extremely concerned	64 (19.11)	115.42 ± 15.30		
Frequency of receiving medication safety education			15.937	<0.001
Frequently	53 (15.82)	111.91 ± 15.93		
Occasionally	157 (46.87)	101.18 ± 16.21		
Rarely/None	125 (37.31)	97.11 ± 15.71		
Number of chronic diseases			0.930	0.426
2	231 (68.96)	101.06 ± 16.66		
3	79 (23.58)	101.06 ± 17.39		
4	16 (4.78)	102.00 ± 12.30		
≥5	9 (2.68)	110.44 ± 17.54		
Disease duration (years)			0.413	0.744
<1	22 (6.57)	103.18 ± 21.81		
1 ~ <5	90 (26.87)	99.83 ± 15.92		
5 ~ <10	106 (31.64)	102.12 ± 13.79		
≥10	117 (34.92)	101.50 ± 18.59		
Disease knowledge level			61.482	<0.001
Not at all knowledgeable	19 (5.67)	76.47 ± 12.14		
Somewhat knowledgeable	172 (51.34)	96.45 ± 13.30		
Very knowledgeable	127 (37.91)	108.28 ± 13.96		
Extremely knowledgeable	17 (5.08)	127.12 ± 11.81		
Years of medication (years)			0.230	0.875
<1	24 (7.16)	101.96 ± 20.37		
1 ~ <5	91 (27.16)	100.25 ± 16.07		
5 ~ <10	109 (32.54)	101.32 ± 13.98		
≥10	111 (33.14)	102.17 ± 18.80		
Number of medications (types)			4.367	0.013
1 ~ 2	87 (25.98)	102.92 ± 19.06		
3 ~ 4	124 (37.01)	97.90 ± 14.40		
≥5	124 (37.01)	103.72 ± 16.59		
Frequency of medication (times/day)			6.652	0.001
1	65 (19.40)	102.40 ± 18.75		
2	149 (44.48)	104.35 ± 17.02		
≥3	121 (36.12)	97.12 ± 14.14		
Has a drug-related adverse event occurred			0.306	0.760
No	244 (72.84)	101.53 ± 16.94		
Yes	91 (27.16)	100.90 ± 16.05		

### 3.3 Scores for participation in medication safety behaviors, health literacy, medication belief, family function, and social network among older adult patients with chronic disease comorbidities

In this study, older adult patients with chronic comorbidities scored 101.36 ± 16.68 for participation in medication safety behaviors, with the lowest average score (2.85 ± 0.72) observed in the medication decision-making dimension. 29.22 ± 5.74 for health literacy, 31.91 ± 5.14 for medication belief, 5.61 ± 1.59 for family function, and 28.48 ± 6.85 for social network (see Table 2).

**Table 2:** Scores for participation in medication safety behaviors, health literacy, medication belief, family function, and social network among older adult patients with chronic disease comorbidities.

Items	Number of entries	Scoring range	Score	Entries evenly distributed
Participation in medication safety behaviors	33	33 ~ 165	101.36 ± 16.68	3.07 ± 0.51
Medication knowledge	12	12 ~ 60	36.56 ± 7.20	3.05 ± 0.60
Pharmaceutical belief	6	6 ~ 30	20.73 ± 3.49	3.46 ± 0.58
Participation in medication decisions	5	5 ~ 25	14.23 ± 3.62	2.85 ± 0.72
Medication self-management	10	10 ~ 50	29.83 ± 5.53	2.98 ± 0.55
Health literacy	10	10 ~ 50	29.22 ± 5.74	2.92 ± 0.57
Medication belief	10	10 ~ 50	31.91 ± 5.14	3.19 ± 0.51
Family function	5	0 ~ 10	5.61 ± 1.59	1.12 ± 0.32
Social network	8	0 ~ 64	28.48 ± 6.85	3.56 ± 0.86

### 3.4 Univariate analysis of participation in medication safety behaviors among older adult patients with chronic comorbidities

Univariate analysis revealed statistically significant differences (*p* < 0.05) in participation in medication safety behaviors scores among older adult patients with chronic comorbidities based on marital status, number of children, education level, place of residence, medical payment methods, average monthly family income, living situation, drug concerns, frequency of receiving medication safety education, disease knowledge level, number of medications and frequency of medication (see Table 1).

### 3.5 Correlation analysis results between participation in medication safety behaviors and health literacy, medication belief, family function and social network among older adult patients with chronic co-morbidities

Correlation analysis revealed that the total score and individual dimension scores of participation in medication safety behaviors among older adult patients with chronic comorbidities were positively correlated with the total scores of health literacy, medication belief, family function, and social network (*r* = 0.347–0.703, *p* < 0.01) (see Table 3).

**Table 3:** Correlation analysis results between participation in medication safety behaviors and health literacy, medication belief, family function and social network among older adult patients with chronic co-morbidities (r).

Items	Participation in medication safety behaviors	Medication knowledge	Pharmaceutical belief	Participation in medication decisions	Medication self-management
Health literacy	0.684**	0.682**	0.383**	0.528**	0.588**
Medication belief	0.639**	0.563**	0.673**	0.347**	0.541**
Family function	0.703**	0.613**	0.596**	0.471**	0.637**
Social network	0.451**	0.407**	0.384**	0.356**	0.354**

**p<0.01.

### 3.6 Multivariate analysis of participation in medication safety behaviors among older adult patients with chronic co-morbidities

Using the total score for participation in medication safety behaviors as the dependent variable, we conducted a multiple stepwise linear regression analysis (α_in_ = 0.05, α_out_ = 0.10) with statistically significant variables from univariate and correlation analyses as independent variables. Multicollinearity diagnosis revealed tolerance coefficients ranging from 0.449 to 0.959 and variance inflation factors from 1.043 to 2.228 across models, indicating no multicollinearity among independent variables. Multivariate linear regression results indicate that education level, drug concerns, disease knowledge level, health literacy, medication belief, family function, and social network entered the regression equation (*p* < 0.05), explaining 75.5% of the total variance (see Table 4).

**Table 4:** Multivariate analysis of participation in medication safety behaviors among older adult patients with chronic disease comorbidities.

Variables	*B*	SE	*β*	95%*CI*	*t*	*p*
Constant	−1.449	6.220	–	(−13.687, 10.789)	−0.233	0.816
Education level	1.689	0.766	0.077	(0.181, 3.197)	2.204	0.028
Drug concerns	2.445	0.609	0.144	(1.247, 3.643)	4.016	<0.001
Disease knowledge level	3.749	0.854	0.153	(2.068, 5.430)	4.388	<0.001
Health literacy	0.806	0.109	0.277	(0.591, 1.021)	7.371	<0.001
Medication belief	0.755	0.114	0.245	(0.531, 0.978)	6.637	<0.001
Family function	2.753	0.386	0.263	(1.993, 3.514)	7.126	<0.001
Social network	0.272	0.075	0.112	(0.124, 0.419)	3.617	<0.001

Independent variable coding method: Education level (Elementary and below = 1, Junior = 2, High school and above = 3); Drug concerns (Not at all concerned = 1, Somewhat concerned = 2, Very concerned = 3, Extremely concerned = 4); Disease knowledge level (Not at all knowledgeable = 1, Somewhat knowledgeable = 2, Very knowledgeable = 3, Extremely knowledgeable = 4).

*R*^2^ = 0.767, adjusted *R*^2^ = 0.755, *F* = 65.246, *p* < 0.001.

## 4 Discussion

### 4.1 Current status of participation in medication safety behaviors among older adult patients with chronic co-morbidities

The results of this study indicate that older adult patients with chronic disease comorbidity in China scored 101.36 ± 16.68 on participation in medication safety behaviors, with an average item score of 3.07 ± 0.51, reflecting a moderate level. However, this score remains lower than that reported by Chinese researchers Liu et al. (29) in their survey of older adult patients with chronic diseases. This discrepancy may be attributed to the increased complexity of medication observed in the study population, which consisted of older adult patients with comorbid chronic diseases. The inclusion of older adult patients with multiple chronic conditions in this study highlights that this population faces greater uncertainties when engaging in medication safety behaviors. Factors such as cognitive decline, impaired hearing or vision, and polypharmacy may increase the risk of missed or incorrect dosing, thereby reducing their actual participation levels (30). Furthermore, long-term management of multiple chronic conditions not only intensifies psychological and financial burdens but also increases the potential for adverse drug interactions, potentially worsening physiological function and diminishing willingness to engage in medication safety behaviors (12).

Analysis of scale dimension scores revealed the lowest scores in the ‘Participation in medication decisions’ dimension. Participation in medication decisions encompasses behaviors such as proactively informing healthcare providers about health and medication status, actively seeking understanding of treatment plans and their benefits/risks, and expressing personal medication needs and treatment expectations (22). The low score in this dimension may be related to the fact that over 80% of patients in this study had an educational attainment of junior high school or below, and nearly 50% were from rural areas. These factors may contribute to limited health literacy, resulting in relatively insufficient awareness and capacity for active participation in treatment decisions (31). Therefore, healthcare providers should prioritize enhancing patients’ capacity to participate in medication decisions when improving medication adherence among those with chronic comorbidities. Based on the COM-B model, to enhance “capability,” structured medication lists and visual aids—including drug names, purposes, dosages, side effects, and notes—can be provided. Systematically implement a “feedback method” process: after medication counseling, ask patients to restate key information in their own words to ensure genuine comprehension and mastery, thereby strengthening knowledge retention and application skills. Second, to create “opportunities,” family-inclusive medication counseling can be implemented. With the patient’s informed consent, a primary family member is invited to participate in the medication education session. Family members are explicitly guided on how to provide effective support (e.g., reminding the patient to take medication, observing reactions rather than substituting decision-making), thereby transforming family dynamics into tangible social support resources. This fosters social and environmental conditions conducive to patient engagement. Additionally, to stimulate “motivation,” design and distribute health education materials—such as short videos or brochures—aimed at reshaping patients’ role identity. These materials should convey the concept that “patients are central to medication safety,” enhancing their sense of self-efficacy and responsibility in decision-making. This approach drives behavioral change at both the cognitive and attitudinal levels, thereby comprehensively improving medication safety behaviors among older adult patients with chronic diseases.

### 4.2 Factors influencing participation in medication safety behaviors among older adult patients with chronic co-morbidities

#### 4.2.1 COM-B model—capacity

In the COM-B model, “Capacity” refers to the physical and psychological knowledge and skills required for an individual to engage in a specific behavior (14). The findings of this study indicate that education level is a significant factor influencing participation in medication safety behaviors among older adult patients with chronic comorbidities, consistent with previous research (32). Patients with higher education level typically possess stronger information comprehension and processing abilities, enabling them to more accurately grasp specialized information such as drug names, dosages, administration schedules, and adverse reactions. Additionally, they are more likely to proactively seek medication knowledge through diverse channels, such as the internet, health manuals, and medical consultations, thereby engaging more actively in medication decision-making and self-management behaviors (33). Therefore, healthcare providers should prioritize low-literacy older adult patients with chronic comorbidities. They can actively develop and promote intuitive medication guidance tools featuring visual aids, videos, and audio explanations—such as medication flowcharts, daily pill organizers, and short instructional videos—to optimize the delivery of complex medication information. Building on this foundation, one-on-one medication education and family involvement in guidance can enhance patients’ ability to correctly understand and apply health information, boosting their confidence and sense of efficacy in self-management. This fosters a virtuous cycle of safe medication use—from identification and comprehension to adherence and ultimately health improvement.

The findings of this study indicate that disease knowledge level is a factor influencing participation in medication safety behaviors among older adult patients with chronic comorbidities. Patients with higher disease knowledge levels are more likely to exhibit proactive participation in medication safety behaviors, consistent with previous research (34). Disease knowledge refers to the extent to which patients understand the causes, symptoms, consequences, and treatment plans of their conditions. It serves as a crucial foundation for influencing treatment adherence and the importance placed on therapy, falling under the “Capacity” dimension of the COM-B model. Typically, patients with higher disease awareness levels can more clearly recognize the potential health risks associated with their condition and more effectively weigh the benefits of treatment against potential barriers. This enables them to participate more proactively in medication adherence and self-management processes (13). Therefore, enhancing disease awareness among patients with chronic diseases is a key pathway to promoting their active engagement in medication safety behaviors. It is recommended to establish a three-tiered collaborative health education model that links hospitals, communities, and households to systematically and multilaterally strengthen the dissemination of knowledge related to chronic diseases. For instance, hospitals can regularly conduct medication safety lectures, communities can organize chronic disease self-management group activities, and households can enhance home medication supervision and support capabilities through caregiver training. Through this tripartite collaboration, patients and caregivers can collectively strengthen their understanding of the disease, build confidence in self-management, and promote long-term medication adherence, thereby establishing a sustainable and effective health behavior support system.

The findings of this study indicate that health literacy is closely associated with participation in medication safety behaviors among older adult patients with chronic comorbidities. Patients with higher health literacy demonstrated greater participation in medication safety behaviors, consistent with previous research (11). Health literacy refers to an individual’s ability to access, understand, process, and act upon essential health information and services to make informed decisions about their health (15). It falls under the “capacity” dimension of the COM-B model. As a vital capacity for maintaining and promoting health, patients with higher health literacy levels typically exhibit greater self-efficacy and health awareness. They can more effectively understand and utilise healthcare resources, thereby proactively participating in medication safety behaviors. Additionally, patients with high health literacy are more inclined to adopt health-promoting behaviors, actively seek and master disease and medication knowledge, enhance self-management capabilities, and more readily comprehend and practice specific behaviors required for medication safety. This significantly enhances their ability to participate in medication safety practices (35). Therefore, healthcare providers are advised to implement multi-tiered, diverse health education activities—such as utilising illustrated materials, short videos, and interactive workshops—to enhance patients’ ability to comprehend and apply health information. Simultaneously, through individualized counseling and medication guidance, we enhance patients’ understanding and acceptance of treatment plans, cultivate their awareness and skills in actively managing their medications, gradually improve their health literacy, and increase their willingness and ability to engage in medication safety practices.

#### 4.2.2 COM-B—opportunity

In the COM-B model, “opportunity” refers to all factors in an individual’s external environment that facilitate the occurrence of behavior, encompassing both physical and social environments (14). This study reveals that family functioning is closely associated with participation in medication safety behaviors among older adult patients with chronic comorbidities, serving as a significant determinant of their engagement in safe medication practices. Family functioning encompasses the support, communication, supervision, and emotional comfort provided by the family, constituting the support within an individual’s immediate social environment (17). This aligns with the “opportunity” dimension in the COM-B model. Family functioning theory posits that families provide essential environmental conditions for their members’ healthy development, encompassing physiological, psychological, and social adaptation (36). Strong family functioning not only helps maintain the physical and mental health of its members but also provides sustained support and assurance for promoting healthy behaviors. Specifically, clear, open, and mutually respectful family communication forms a crucial foundation for safe medication practices. Effective family care helps alleviate anxiety, frustration, and helplessness stemming from chronic co-morbidities and polypharmacy (31). When patients experience missed doses, medication errors, or suspected adverse drug reactions, family members can promptly identify risks, assess situations, and implement appropriate interventions (37). Therefore, while healthcare providers focus on patients’ disease-related physical and psychological health issues, they should also prioritize assessing family functioning and actively mobilize the family support system. This can be achieved through family meetings, care guidance, and encouraging shared participation in health decisions, thereby guiding family members to provide positive emotional support.

The findings of this study indicate that social networks are a significant factor influencing participation in medication safety behaviors among older adult patients with chronic comorbidities. Specifically, higher levels of social network engagement correlate with greater adherence to medication behaviors, consistent with previous research (38). A social network refers to the aggregate of an individual’s interactive relationships with family members, friends, relatives, and other social contacts (18). A robust social network provides individuals with ample resources, information, and emotional support, falling under the “Opportunities” dimension of the COM-B model. Social network theory posits that populations with differing characteristics form distinct network structures due to variations in internal communication methods and information transmission patterns (39). These structures directly influence knowledge dissemination pathways and effectiveness, thereby profoundly affecting individual mental health and behavioral choices. A robust social network provides patients with multifaceted emotional support from family and friends, helping to bolster their confidence in coping with illness and the treatment process. Simultaneously, social networks function as “filters” and “converters” for medical information. They help patients comprehend medication instructions, recognize adverse drug reactions, or gain practical treatment insights through peer group exchanges, thereby reducing misinformation and improving information utilisation efficiency (40). Previous studies have also indicated that higher social network levels positively influence the psychological well-being and disease management capabilities of patients with chronic diseases (41). Therefore, healthcare providers should prioritize assessing the social network status of older adult patients with chronic comorbidities and actively strengthen community support systems to better address their needs. This includes promoting the establishment of community medication management teams and peer support programs for chronic conditions, while encouraging patients to actively participate in community-organized health lectures, self-management training, and similar activities. Simultaneously, healthcare providers should actively encourage family members and friends to become more involved in daily medication reminders, emotional support, and accompanying patients during medical visits. This approach systematically enhances the quality and utilization of patients’ social support networks, thereby establishing a robust social foundation for sustaining safe medication practices.

#### 4.2.3 COM-B model—motivation

In the COM-B model, “motivation” refers to all brain-based psychological processes driving behavior. It encompasses both conscious planning and unconscious emotions and impulses (14). This study reveals that drug concerns has a significant influence on participation in medication safety behaviors among older adult patients with chronic comorbidities. Drug concerns reflects a person’s focus on drug-related information. It represents an active, conscious process of reflection and focus that drives information-seeking and shapes medication decisions (42). It is a form of reflective motivation. Typically, patients with higher drug concerns are more inclined to proactively seek medication-related information (such as package inserts, drug interactions, and adverse reactions) and effectively translate such information into practical knowledge, thereby maintaining good medication adherence. Additionally, patients with higher drug concerns place greater emphasis on obtaining professional information from authoritative sources, such as physicians and pharmacists, and actively participate in their own medication decisions, which consequently demonstrates higher medication self-management capabilities (5). Therefore, enhancing patients’ awareness of the benefits of drug therapy is a crucial step in improving their engagement in medication safety behaviors. This suggests that healthcare providers should actively conduct personalized medication education. By clearly explaining the goals, expected outcomes, and potential risks associated with drug therapy, healthcare providers can help patients establish realistic expectations about medication use. Simultaneously, encouraging patients to ask questions and express concerns during consultations strengthens their willingness and ability to participate in medication decisions, thereby improving medication safety levels systematically.

The findings of this study indicate that medication beliefs are positively correlated with participation in medication safety behaviors among older adult patients with chronic comorbidities. Specifically, stronger medication beliefs correlate with more active participation in medication safety behaviors, consistent with previous research (43). Medication beliefs refer to patients’ perceptions of the necessity of drug therapy and their concerns about potential adverse reactions, reflecting individual cost–benefit considerations in medication decisions (16). This corresponds to the “motivation” dimension within the COM-B model. As a subjective cognitive construct, medication beliefs are influenced by multiple factors, including family, society, environment, and disease perception, and have a direct impact on treatment health outcomes and healthcare costs. Patients with chronic disease comorbidities typically experience prolonged treatment cycles, recurrent symptoms, and significant psychological burdens, making them more prone to developing apprehensive medication beliefs. This can lead to non-adherent behaviors such as arbitrarily reducing or discontinuing medication (44). Research indicates that stronger beliefs in the necessity of medication are correlated with reduced concerns about potential adverse effects, leading to improved treatment adherence and a greater willingness to engage in safe medication practices (45). Therefore, healthcare providers should enhance targeted health education for patients with chronic comorbidities, helping them overcome misconceptions and anxieties about their conditions and drug therapies while strengthening their conviction in the necessity of medication. For patients who have not yet developed a firm belief in medication adherence, case-based teaching methods can be employed. Real-life examples help them deeply recognize the risks of arbitrarily discontinuing medication or altering treatment regimens. Additionally, continuous health guidance and support should be provided based on patients’ healthcare needs, strengthening their self-management capabilities and medication adherence throughout the entire disease course.

## 5 Limitations

This study also has certain limitations. First, the investigation was conducted exclusively among older adult patients with chronic disease comorbidities at a Grade A tertiary hospital in Sichuan Province, China. This limits the representativeness and generalizability of the sample. Grade A tertiary hospitals typically treat patients with more complex conditions and a higher number of comorbidities. Therefore, our sample may not adequately represent patients with relatively stable chronic diseases who receive care at community health service centers or primary care facilities. These patients may differ systematically in socioeconomic status, health literacy, disease severity, and access to medical resources. Future multi-center, large-sample studies should include patients from various medical institutions (such as community hospitals and secondary hospitals) for comparative analysis. This approach could provide a more comprehensive exploration of medication adherence behaviors among older adult patients with chronic disease comorbidities, considering different regions, economic levels, and hospital tiers. Second, this cross-sectional study cannot establish causal relationships between variables. Its findings reflect only the participants’ cognitive and behavioral levels at the time of the survey, without assessing longitudinal changes. Future longitudinal studies are needed to analyze dynamic shifts in medication adherence behaviors among older adult patients with chronic comorbidities. Additionally, the study relies primarily on patient self-reports, which may be affected by social desirability and recall biases. Although anonymous surveys and standardized instructions were used to reduce these biases, future research should include objective indicators for validation. Finally, the study did not measure clinician-level factors such as the quality of doctor-patient communication, prescribing habits, or attitudes toward patient involvement. Future research should integrate objective clinical data with patient-reported outcomes and add cognitive screening of participants to more fully evaluate patient engagement in medication safety behaviors.

## 6 Conclusion

In summary, the level of participation in medication safety behaviors among Chinese older adult patients with chronic comorbidities remains moderate, indicating significant room for improvement. This participation in medication safety behaviors is influenced by multiple factors, including capability, opportunity, and motivation, with health literacy, medication belief, family function, and social network emerging as key determinants. These findings suggest that healthcare providers should develop targeted intervention strategies tailored to the characteristics and needs of older adult patients with chronic comorbidities to enhance their participation in medication safety behaviors.

## Data Availability

The datasets presented in this study can be found in online repositories. The names of the repository/repositories and accession number(s) can be found in the article/Supplementary material.
